# “There’s No Heroin Around Anymore. It’s All Fentanyl.” Adaptation of an Opioid Overdose Prevention Counseling Approach to Address Fentanyl Overdose: Formative Study

**DOI:** 10.2196/37483

**Published:** 2022-09-07

**Authors:** Vanessa M McMahan, Justine Arenander, Tim Matheson, Audrey M Lambert, Sarah Brennan, Traci C Green, Alexander Y Walley, Phillip O Coffin

**Affiliations:** 1 San Francisco Department of Public Health San Francisco, CA United States; 2 Grayken Center for Addiction Clinical Addiction Research and Education Unit School of Medicine, Boston University Boston, MA United States; 3 Boston Medical Center Boston University Boston, MA United States; 4 Heller School for Social Policy and Management Brandeis University Waltham, MA United States; 5 Center of Biomedical Research Excellence on Opioids and Overdose Rhode Island Hospital Providence, RI United States; 6 Department of Medicine University of California, San Francisco San Francisco, CA United States

**Keywords:** opioid overdose, fentanyl, motivational interviewing, naloxone, assessment, decision, adaptation, production, topical experts, integration, training, and testing, ADAPT-ITT, theater testing

## Abstract

**Background:**

Drug overdose mortality continues to increase, now driven by fentanyl. Prevention tools such as naloxone and medications to treat opioid use disorder are not sufficient to control overdose rates; additional strategies are urgently needed.

**Objective:**

We sought to adapt a behavioral intervention to prevent opioid overdose (repeated-dose behavioral intervention to reduce opioid overdose [REBOOT]) that had been successfully piloted in San Francisco, California, United States, to the setting of Boston, Massachusetts, United States, and the era of fentanyl for a full efficacy trial.

**Methods:**

We used the assessment, decision, adaptation, production, topical experts, integration, training, and testing (ADAPT-ITT) framework for intervention adaptation. We first identified opioid overdose survivors who were actively using opioids as the population of interest and REBOOT as the intervention to be adapted. We then performed theater testing and elicited feedback with 2 focus groups (n=10) in Boston in 2018. All participants had used opioids that were not prescribed to them in the past year and experienced an opioid overdose during their lifetime. We incorporated focus group findings into our initial draft of the adapted REBOOT intervention. The adapted intervention was reviewed by 3 topical experts, and their feedback was integrated into a subsequent draft. We trained study staff on the intervention and made final refinements based on internal piloting. This paper describes the overall ADAPT-ITT process for intervention adaptation, as well as a qualitative analysis of the focus groups. Working independently, 2 authors (VMM and JA) reviewed the focus group transcripts and coded them for salient and common themes using the constant comparison method, meeting to discuss any discrepancies until consensus was reached. Codes and themes were then mapped onto the REBOOT counseling steps.

**Results:**

Focus group findings contributed to substantial changes in the counseling intervention to better address fentanyl overdose risk. Participants described the widespread prevalence of fentanyl and said that, although they tried to avoid it, avoidance was becoming impossible. Using alone and lower opioid tolerance were identified as contributors to overdose risk. Slow shots or tester shots were acceptable and considered effective to reduce risk. Naloxone was considered an effective reversal strategy. Although calling emergency services was not ruled out, participants described techniques to prevent the arrival of police on the scene. Expert review and internal piloting improved the intervention manual through increased participant centeredness, clarity, and usability.

**Conclusions:**

We successfully completed the ADAPT-ITT approach for an overdose prevention intervention, using theater testing with people who use opioids to incorporate the perspectives of people who use drugs into a substance use intervention. In the current crisis, overdose prevention strategies must be adapted to the context of fentanyl, and innovative strategies must be deployed, including behavioral interventions.

**Trial Registration:**

ClinicalTrials.gov NCT03838510; https://clinicaltrials.gov/ct2/show/NCT03838510

## Introduction

### Background

Since 1999, drug overdose deaths have risen 4-fold in the United States, with the majority of overdose deaths related to opioids [1]. We are now in the “third wave” of the overdose epidemic, characterized predominantly by fentanyl and fentanyl-related overdose mortality [2,3]. From 2015 to 2021, the annual number of overdose deaths due to synthetic opioids, which are almost exclusively fentanyl and related analogs, rose 93% from 51,575 to 99,543 [4]. Fentanyl deaths have supplanted heroin deaths; although all opioid deaths have been increasing, heroin-involved deaths that did not also involve fentanyl have decreased [5]. Opioid overdose prevention strategies must be adapted to appropriately address fentanyl overdose.

Providing naloxone to laypeople who may witness an opioid overdose is highly effective at preventing overdose mortality [6-10]; however, the rapidity of fentanyl overdose limits the time frame in which naloxone can be effectively administered. Therefore, additional and complementary opioid overdose interventions are urgently needed [11]. Motivational interviewing has been shown to reduce opioid risk behaviors [12,13]. From 2014 to 2016, we completed a pilot in San Francisco, California, United States, of a repeated-dose motivational interviewing intervention (repeated-dose behavioral intervention to reduce opioid overdose [REBOOT]) aimed at reducing overdose occurrence and mortality among persons who had previously overdosed and had received take-home naloxone. Participants randomized to the REBOOT intervention were less likely to experience an opioid overdose during 16 months of follow-up, and those who did overdose had fewer overdose events [14]. Building on these pilot results, we designed a clinical trial (REBOOT 2.0) to determine the efficacy of the REBOOT intervention on overdose occurrence and number of overdoses (NCT03838510).

### Adapting the REBOOT Counseling Intervention

To address the emergence of fentanyl in eastern US states between the pilot and REBOOT 2.0, as well as to ensure that the intervention was appropriate for a broader geographic region, we sought to adapt the REBOOT counseling intervention using the assessment, decision, adaptation, production, topical experts, integration, training, and testing (ADAPT-ITT) model [15]. We describe the ADAPT-ITT process and focus group findings, highlighting how the perspectives of people who use drugs guided the intervention adaptation process to address fentanyl overdose risk.

## Methods

### ADAPT-ITT Framework

ADAPT-ITT is an effective framework for adapting HIV-prevention evidence-based interventions to new populations and settings, which includes 8 sequential phases to iteratively elicit feedback from the population of interest and key stakeholders [15]. This is the first study to our knowledge to use ADAPT-ITT to adapt an opioid overdose prevention intervention, although it has been used previously to adapt other interventions for people who use opioids to new settings [16,17]. We conducted a modified ADAPT-ITT process to adapt the REBOOT overdose counseling intervention to be appropriate in the context of fentanyl.

### Phases 1 (Assessment) and 2 (Decision)

The aim of phase 1 of the ADAPT-ITT model is to identify the population at risk, and the aim of phase 2 is to identify the appropriate intervention to adapt. We identified people with overdose history as the population at risk, considering their elevated risk for opioid overdose [18], and chose to adapt the REBOOT counseling intervention based on its success during our pilot [14]. Therefore, we modified phases 1 and 2 of the ADAPT-ITT approach to be internal, and we did not review additional overdose reduction interventions for potential adaptation. As these phases were completed before project initiation, they are not described further herein.

### REBOOT Counseling Intervention

The REBOOT counseling intervention aimed to engage participants in a discussion of their opioid overdose history, both witnessed and experienced; discuss overdose risk behaviors; and identify feasible risk reduction strategies that the participant would be interested in using. Specifically, the 6 counseling steps are as follows: (1) explore participants’ experiences witnessing an overdose and contributing factors, (2) discuss participants’ own most recent overdose and contributing factors, (3) review overdose risk behaviors, (4) review ways to reduce overdose risk, (5) discuss participants’ current and additional planned strategies to reduce overdose risk and provide them with a wallet card listing risk-reduction strategies, and (6) review 3 steps in the Skills and Knowledge on Overdose Prevention (SKOOP) Project overdose recognition and reversal curriculum (ie, recognize an overdose, respond to an overdose, and provide aftercare) [19]. In addition, during the session, the counselor provides and reviews with participants 3 handouts that describe activities that increase opioid overdose risk, ways to reduce risk, and steps to respond to a witnessed overdose. REBOOT is intended for individuals who have previously received take-home naloxone, but we do not distribute naloxone as part of the counseling intervention.

### Phase 3 (Administration)

#### Overview

In phase 3 of ADAPT-ITT, the intervention is theater tested, and feedback from the audience is elicited. Theater testing involves inviting individuals of the population at risk to watch a demonstration of the intervention and then answer questions about their opinions of the intervention. On September 26, 2018, we conducted theater testing with 2 focus groups in Boston, Massachusetts, United States. REBOOT had been piloted in San Francisco, and at that time there was very little fentanyl being used in that city; thus, the adaptation effort was focused on Boston, where fentanyl was the dominant opioid [20-22].

#### Focus Group Participants

We contacted individuals who had reported opioid use while participating in previous studies at the Boston Medical Center and provided consent for future contact for research. Eligibility criteria for the focus groups included age 18 to 65 years, living in the Boston metropolitan area, using an opioid in the past year that was not prescribed, lifetime history of overdose, and comfort participating in the focus group in English. Participants who were screened for eligibility reported their demographic characteristics (ie, age, gender, race, and ethnicity), overdose history, lifetime naloxone use, and past-year opioid use. Participants reviewed an information sheet with study staff and provided verbal consent for participation.

#### Focus Group Procedures

In each focus group, participants were shown the same video of a counseling session with a participant from San Francisco. The counseling session lasted 39 minutes and was conducted by a trained clinical psychologist with expertise in motivational interviewing following the REBOOT counseling steps. The handouts provided to the participant at the counseling session were also provided to focus group participants for review. A video recording of the session was used, rather than a live demonstration, because of the short-acting nature of fentanyl and the risk that focus group participants would develop opioid withdrawal symptoms if there were delays in the procedures.

During the viewing of the video and afterward, participants were asked questions following a semistructured interview guide about their opinions and perspectives regarding the counseling session (Multimedia Appendix 1). At the end of each focus group, participants were given US $40 for their participation. Focus groups were audio recorded and transcribed using Rev software [23].

#### Focus Group Analysis

Working independently, 2 authors (VMM and JA) reviewed the focus group transcripts and coded them for salient and common themes using the constant comparison method [24]. A list of a priori codes of common themes related to opioid overdose was used to help guide transcript coding (eg, “access to naloxone,” “tester shot,” and “calling 911”). VMM and JA met after coding each focus group transcript to compare codes and discuss them until they reached consensus. As codes emerged as focus group transcripts were reviewed, both focus group transcripts were reviewed a second time after the final list of codes was determined. Codes and themes were then mapped onto the 6 steps of the REBOOT counseling intervention. All transcript coding and analyses were performed using ATLAS.ti software (version 8; ATLAS.ti Scientific Software Development GmbH).

### Ethics Approval

This study was approved by the University of California San Francisco Institutional Review Board (17-24203).

### Phases 4 (Production), 5 (Topical Experts), and 6 (Integration)

Focus group findings were used to produce a draft of the adapted counseling intervention (phase 4), which was reviewed by 3 topical experts (phase 5). The experts were overdose education and prevention professionals from the Boston Public Health Commission, Harm Reduction Coalition, and the Education Development Center. The expert feedback was integrated into the subsequent draft of the counseling intervention (phase 6).

### Phases 7 (Training) and 8 (Testing)

We then trained our counseling staff (phase 7) and tested the adapted intervention (phase 8) to make final refinements. We modified phase 8 of ADAPT-ITT by conducting internal monitoring with the study team and not a pilot with the population of interest. The final adapted counseling intervention is being evaluated in the ongoing REBOOT clinical trial.

## Results

### Phase 3: Focus Group Findings

In total, 21 individuals were eligible and invited to participate in the focus groups. Of these 21 individuals, only 10 (48%) attended the focus groups, with 6 (60%) in one focus group and 4 (40%) in the other. The mean age of participants was 44.1 (SD 13.8) years, and the majority were men (7/10, 70%). Half (5/10, 50%) of the sample was Hispanic/Latinx; in terms of race, 20% (2/10) were Black or African American, 30% (3/10) were White or Caucasian, and 50% (5/10) reported another race. All participants had experienced an overdose involving fentanyl, and 90% (9/10) had used fentanyl in the past year (Table 1).

**Table 1:** Participant demographic characteristics and experience with fentanyl (N=10).

Demographic characteristics		Values
**Number of participants, n (%)**		
	Focus group 1	6 (60)
	Focus group 2	4 (40)
Age (years), mean (SD)		44.1 (13.8)
**Ethnicity, n (%)**		
	Non-Hispanic/Latinx	5 (50)
	Hispanic/Latinx	5 (50)
**Race, n (%)**		
	Black or African American	2 (20)
	White or Caucasian	3 (30)
	Other race	5 (50)
**Gender, n (%)**		
	Male	7 (70)
	Female	3 (30)
**Fentanyl, n (%)**		
	Used fentanyl in the past year	9 (90)
	Experienced an overdose involving fentanyl	10 (100)

### Step 1: Explore Participants’ Experiences Witnessing an Opioid Overdose, Factors Contributing to This Overdose, and Thoughts About Own Substance Use Since This Witnessed Overdose

All participants reported that they had witnessed an opioid overdose. Participants described various strategies to respond to a witnessed overdose, including using naloxone, calling 911, and other strategies stemming from their lived experience.

#### Naloxone

All participants had previously received take-home naloxone and were generally knowledgeable about how to use naloxone. In total, 80% (8/10) of the participants reported administering naloxone to someone who was overdosing:

Narcan [a brand name for naloxone]...I have that in my house for my son. I have a ring and it shows you what to do...

Focus group 1, attendee 2

#### Calling 911

Participants thought that calling 911 had inherent risks, including arrest and loss of housing. Strategies to mitigate these risks included relying on the Good Samaritan law, although there was discussion regarding how it only protected the individual calling 911 from arrest, and not others present at the scene of an overdose. Participants also recommended not telling a dispatcher that the person had overdosed, but instead saying that they were “not breathing” or “unresponsive” so that medical services would arrive without the police:

A lot of people are afraid to call the cops [for an overdose] because they think they’re going to get locked up. I’ve seen people like, “Leave him, leave him. Let’s go.”

Focus group 1, attendee 5

I’m not trying to be a jerk, but you pull [the person who overdosed] out in the hallway and [tell 911], “Listen. I came outside, and there’s somebody unconscious on my ground. Is it drug-related? I don’t know. Here’s the address.” And hang up quick so they can make it there quick. [Focus group 1, attendee 1)

#### Additional Overdose Response Strategies

Participants described several strategies to reverse a witnessed opioid overdose that stemmed from their lived experience. These included injecting the individual who had overdosed with salt and water or cocaine and placing the individual in an ice bath or putting ice on their genitals, which participants emphasized they had seen be successful:

They shoot you with salt. They put ice on your testicles. Water...They just inject the salt, and it gives you a reaction without being in the vein, then it wakes you up. I have seen it with my own eyes, people got woken up with a shot of salt. They say it doesn’t work but I have seen it work.

Focus group 1, attendee 4

### Step 2: Discuss Participants’ Own Most Recent Overdose and Contributing Factors

All focus group participants reported experiencing an opioid overdose during their lifetime, consistent with eligibility criteria, and all participants reported a lifetime overdose that involved fentanyl. Among those who described their overdose experiences, most had been administered naloxone to reverse the overdose. A lower opioid tolerance after being released from jail or leaving treatment was commonly identified as a contributing factor for opioid overdose:

I had just come out of jail three weeks prior. I was already using dope but what I was doing was I was using tester shots. If I had a 30 for myself, I would do a 15 or like a 10 [to] see how it made me feel. If it made me feel alright, I would just save the other shot, because, at the end of the day, you can only just do more. I looked at my girlfriend, I’m like “Fuck that I’m going to do the whole 30.” I did it and I felt the heat from my toes up I was like “Damn that shit was strong.” Next thing I remember, I’m holding onto a fence, I’m like “What the hell?” I'm looking around, she’s crying. I’m like “What the hell happened?” She’s like you’ve been dead for like seven minutes. I’m like “Oh my god.” She had just woke me up. They Narcan-ed me like three times, sternum rubbed me and when they threw water on me, I guess that’s when I responded.

Focus group 1, attendee 6

### Step 3: Review Behaviors That Increase Opioid Overdose Risk

#### Overview

In step 3 of the counseling session, the counselor provided a handout to the participant listing several behaviors that increase the risk for an overdose (Multimedia Appendix 2). The counselor read the list out loud with the participant and then explored the participant’s own risk for overdose. After viewing the counseling session and reading the handout, the focus group participants highlighted 3 main risk factors for opioid overdose: fentanyl, opioid tolerance, and duration of opioid use.

#### Fentanyl

Participants across both focus groups described fentanyl as prevalent and a major contributor to opioid overdose:

They [are] even putting [fentanyl] on the weed. On the cocaine...

Focus group 1, attendee 4

There’s no heroin around anymore. It’s all fentanyl.

Focus group 2, attendee 4

Participants also described how fentanyl was being mixed with other drugs unbeknownst to those using those drugs:

They’re putting [fentanyl] in the coke now, the cocaine. My friend [who] only does coke...when he stopped doing coke, he was like dope sick. He don’t do dope or nothing and had all the symptoms of dope sickness and then—it’s a couple—when she did her test at the methadone clinic, she was coming up for fentanyl and she was only doing coke.

Focus group 1, attendee 2

#### Opioid Tolerance

Lower opioid tolerance after not using opioids for a period of time, in particular after being in jail or substance use disorder treatment, was identified as a risk factor for overdose:

Every time someone just get[s] out [of jail or treatment] they think they can do the same thing as when they went in...[but] they’ll automatically OD.

Focus group 2, attendee 1

#### Duration of Opioid Use

Focus group participants described overdose risk as higher among people who had recently begun using opioids. Participants frequently described themselves as people who had used opioids for a long period of time and therefore were at less risk of overdose. The median length of time since first using opioids that were not prescribed was 26.5 (IQR 17-35) years. A participant described the utility of counseling among those starting to use opioids:

I think [overdose counseling] would be helpful for kids who are just starting using, who aren’t like smart into the lifestyle and know the game...who are more reckless than a lot of us who have been using for years.

Focus group 2, attendee 4

Although participants described their duration of opioid use as lowering their overdose risk during the focus groups, on the screening questionnaire, of the 10 participants, 7 (70%) said that they felt their risk of overdose was high, 2 (20%) said that it was moderate, and 1 (10%) reported low risk. The participant who reported low overdose risk had used opioids for the shortest period of time (5 years). Thus, although participants who had used opioids longer thought that their overdose risk was lower than people starting to use opioids, they still reported a moderate to high risk for overdose.

### Step 4: Review Ways to Reduce Overdose Risk

In step 4 of the counseling session, the counselor provided another handout to the participant that listed several strategies that could be used to reduce one’s risk of opioid overdose (Multimedia Appendix 2). After watching the counseling session and reading the corresponding handout, focus group participants discussed 5 main strategies to reduce the risk of opioid overdose: not using alone, avoiding fentanyl, using the same dealer, using less or using partial shots or tester shots, and medications for opioid use disorder (MOUD).

#### Do Not Use Alone

There was consensus that not using alone was an effective way to lower the risk of opioid overdose, and most participants reported that they usually use with others, and when they use alone, they may call someone, use in a bathroom in a monitored environment (eg, community-based organization), or make sure a friend can access them:

I try to hang out with friends, I don’t use by myself now. It’s like you kind of do it in a group, you know? So, each know each other’s tolerances.

Focus group 1, attendee 3

When I do [opioids] by myself in my house, I leave the doors unlocked, you know? I got a good friend of mine that has a copy of a key. That’s the only thing that I do [for overdose prevention].

Focus group 2, attendee 5

#### Avoid Fentanyl

Across both focus groups, participants discussed how they aimed to avoid fentanyl because of its prevalence and potency; however, they considered avoiding fentanyl increasingly difficult because of the rapidly rising prevalence of fentanyl. Ways to identify fentanyl included its gray color; sweet taste; and smell, which is different from that of heroin. Using fentanyl test strips, as suggested in the counseling video, were discussed but were not considered accessible to focus group participants in Boston at the time:

[I do] a whole lot [differently because of fentanyl in the drug supply], make sure you don’t get that bullshit. Go to somebody you know that’s got real dope.

Focus group 2, attendee 3

Yeah, a lot of them [lie and] say “No, it’s straight dope, it’s straight dope. There’s no fentanyl.” That's why a lot of people they’re doing pills. They rather be doing pills than do the dope.

Focus group 1, attendee 6

Participants also described a paradox, whereby although they typically tried to avoid fentanyl, if they learned of strong opioids, they wanted to use them—they sought them out:

*Some people would see somebody overdose and be like, yo, who did he buy that from?...Because if it puts you out then it means it’s some good shit.So other people be like, yo, where do you buy that from? I want some of that shit.* [Focus group 1, attendee 3]

*If over there in the corner, somebody comes and tells me...”that shit over there is killing motherfuckers,” I’m going to go over there, excuse my language, because there’s some good dope over there. We never learn. You understand? I’ll ask who’s got the good stuff, “that one’s killing people be careful” and I’ll go over there.* [Focus group 1, attendee 4]

One way that participants rationalized this paradox was that they thought that they personally would not overdose from drugs that others had overdosed from because they had a higher tolerance:

We don’t think we’re going to OD off of it. We think like, “Oh, my habit’s worse than your habit...” I have a 500-dollar-a-day habit.

Focus group 2, attendee 4

#### Using Less or Using Partial Shots or Tester Shots

Participants discussed strategies to reduce the amount of drugs used or sample drugs before using them, through either partially depressing the plunger before injecting all of the solution (*slow shot*) or doing a tester shot. Participants stressed that using less was especially important when fentanyl might be present:

If you know there’s fentanyl in there, instead of doing a 40 you do a 20...It will be harm reduction for myself because if I’m doing a 40 of fentanyl now, and then I come and test it before I do that 40 and it’s got fentanyl, I’m going to do a 20 because the 40 is going to kill me.

Focus group 1, attendee 4

However, there were mixed feelings about partial shots or tester shots, and positive perceptions were generally shared in the context of expected lower tolerance (eg, having recently been incarcerated):

When I’m getting [high], I’m getting [high]. Forget about that partial test.

Focus group 1, attendee 4

I’ve learned that every time I get out of jail I just do less, like a tester shot now. My boyfriend would always say to me, you can always do more, but you can’t take one back. So, stop being a glutton, it’s right here...It stuck in my head like he’s got a point. You can do another one, but you can’t take one back.

Focus group 2, attendee 2

#### Use the Same Dealer

The participant in the counseling video used the same dealer to avoid getting fentanyl. Across both focus groups, participants did not think that using the same dealer was an effective way to avoid fentanyl because of the uncertainty in the drug supply:

[The lady in the video] gets it from the same guy all the time...That don’t mean nothing. The guy doesn’t know what chemicals the drug got. He could say, “It’s good don’t worry about it,” and when she gets home [she overdoses]. You never know what could happen.

Focus group 1, attendee 4

Well, because he could be the same drug dealer, but he might have gotten a different batch. They don’t always get the same thing. There are different grades of fentanyl. There are stronger grades that have legs, there are shit grades that you really get pissed about...

Focus group 2, attendee 2

#### Treatment for Opioid Use Disorder

Although, in line with the participant-centered harm reduction counseling approach, MOUD was included on the handout of possible strategies to reduce the risk of opioid use disorder, it would only be discussed with the participant if they demonstrated interest in MOUD. A few (2/10, 20%) of the focus group participants said that they thought this was not sufficient and that the counseling intervention would be more effective if MOUD was emphasized more:

I think it was helpful to encourage [the participant in the video to stay] clean, but [the counselor in the video] didn’t offer any places like we should try, like methadone maintenance, if you haven’t been able to maintain completely off anything, or Suboxone or halfway house.

Focus group 2, attendee 2

### Step 5: Create or Provide a Wallet Card for Participants Based on Their Current and New Efforts to Reduce Risk of Overdose

Toward the end of the counseling session, the counselor created a wallet card for the participant based on the overdose prevention strategies that the participant described as part of their current practice or additional strategies of interest to them. Focus group participants thought that providing a wallet card to participants to remind them of what was discussed in the counseling session was helpful. Using other forms of technology to remind participants of the discussed strategies was also suggested:

Maybe even, nowadays with technology, it could even be on an iPad...Or text messages, or another app that you can check on.

Focus group 1, attendee 2

Although participants thought that the wallet card was useful, participants recommended providing more handouts, naloxone, and fentanyl test strips, as well as providing training for rescue breathing and naloxone administration:

If you’re actively using, [a brochure about resources is] just a piece of paper, it’s nothing, you know what I mean? But if you’re trying to change your life and get help then like it can be extremely helpful. But it all depends on where you are.

Focus group 2, attendee 4

### Step 6: Review 3 Steps in Management of Witnessed Overdose (SKOOP Curriculum)

The last step was to review a handout that described the 3 steps in management of a witnessed overdose from the SKOOP Curriculum [19]: recognizing an overdose, responding to an overdose, and providing aftercare. Focus group participants commented on how the participant in the video learned additional information about overdose response during this step and that the use of a handout was helpful:

When [the counselor in the video] had it in black and white right in front of her and she was reading and she’s like, “Oh I didn’t know about the recovery position,” so she was very perceptive to it, you know what I mean?...rather than just talking about things, like when it’s in black and white, right in front of you. Like for me, I tend to pick things up by reading them...’cause I’ll forget what you’re telling me...if it’s right in front of me, it’s hard to ignore.

Focus group 2, attendee 4

### Overall Suggestions for the Counseling Session

Focus group participants reported that they already knew a lot of the information that was shared in the counseling session, and they thought that the best audience would be younger people who have not used opioids for very long:

[The counseling session] might be helpful for the people just getting into this game, but I’ve been in this game since ’69, so it might be helpful to the newcomers.

Focus group 2, attendee 1

Some (3/10, 30%) of the participants recommended including a conversation about the root causes of people’s substance use and expanding the intervention to include a broader counseling intervention:

I’ve heard [the content of the counseling session] multiple times. I think I agree with the whole overdose prevention and training in Narcan, reiterating it and whatnot. But I think they could’ve focused on a little bit more of core issues of why people use and why do they continue to go back. Because [the woman in the video] had six months clean in jail. You know what I mean? Like she didn’t have to go back and get high. Like granted nine out of 10 of us are going to. You know what I mean? But like at that point you’re already over the physical part of it. So, like start dealing with your mental side and like those cravings...I think they could like integrate that into a lot of the conversations.

Focus group 2, attendee 4

### Phase 4: Production

We incorporated the focus group findings to adapt the REBOOT counseling intervention to better address fentanyl overdose risk. Specifically, we highlighted avoidance of fentanyl and using tester shots and de-emphasized using the same dealer. We added the recommendation to test drugs for fentanyl and identified locations to refer participants to obtain fentanyl test strips. Counseling staff were alerted that even among those who are aware of fentanyl’s overdose risk there may be a desire to use fentanyl; they were directed to explore this paradox with individuals who use opioids during counseling and tailor overdose prevention strategies as appropriate. Staff were also trained to understand that participants may underestimate their overdose risk, and they were provided guidance on how to help participants develop a realistic personal overdose risk assessment. We also added tips when calling 911 to mitigate the chances of police arriving at the overdose scene.

### Phases 5 and 6: Topical Experts and Integration

We shared a draft of the counseling manual describing the adapted REBOOT intervention informed by the focus groups with 3 overdose prevention and education experts for their review and feedback. All 3 experts sent suggestions for the draft intervention, which were incorporated into the next iteration of the counseling approach.

Recommendations included adding less risky routes of administration as a way to reduce overdose risk (eg, smoking instead of injecting) and asking participants to “walk through” a typical day to visualize how overdose prevention strategies could realistically be implemented in their life, as well as asking participants what they want to “change” about their substance use, not whether they want to “reduce” or “stop” their substance use.

In response to participant and expert feedback, we developed an assessment of the level of interest in substance use treatment and any plans or needs to access substance use treatment, which is conducted in at least one of the 4 REBOOT counseling sessions. Similar to the overdose counseling approach, the substance use treatment counseling component aims to be participant-centered and begins by exploring participants’ previous substance use treatment experience before assessing interest.

### Phases 7 and 8: Training and Testing

After incorporating the expert feedback into the counseling intervention, we trained study site staff by reviewing the manual and handouts, and team members conducted role plays with a trained clinical psychologist. Study staff provided further minor modifications to the approach to best operationalize it. Recommendations included improving readability (eg, using bullets, larger font, and more spacing), including the expected duration of the counseling session at the beginning, and using a more open-ended question when assessing the participants’ personal overdose history (ie, “Now let’s discuss the last time you experienced an overdose. Thinking back to that time, please tell me the story of your most recent overdose”).

## Discussion

### Findings and Contextualization

We adapted an opioid overdose prevention intervention to the era of fentanyl and a broader geographic region through the ADAPT-ITT process. As expected, the prevalence of fentanyl in Boston was the dominant issue for overdose prevention, and the process resulted in multiple adaptations to the final intervention. Focus group participants who used opioids emphasized the importance of fentanyl avoidance (eg, by assessing drug color, taste, and smell), as well as the growing impossibility of avoiding fentanyl. The predominant role of fentanyl in increasing overdose risk has also been reported in other studies among people who use opioids [25-27].

Although participants described the importance and difficulty of avoiding fentanyl, they also described the desire to use drugs that were involved in an overdose because of that indicated potency. This mix of fentanyl avoidance and seeking was also seen in a qualitative study in Pennsylvania, United States, among persons with recent opioid misuse or heroin use [28]. Participants seemed to be aware of this paradox but rationalized it by characterizing themselves as using opioids for a long time and having a high opioid tolerance and, therefore, at lower risk of an overdose. This was similar to our findings among REBOOT pilot participants, where participants who were older had lower odds of perceived overdose risk [29]. Pilot participants similarly described inexperience as a main contributor to overdose events that they witnessed in other people but not to overdoses that they personally experienced [30]. This finding led to a greater emphasis in the intervention on helping participants develop a realistic assessment of their risk for overdose.

The predominant role of fentanyl in opioid overdose affected participants’ perceptions of traditional overdose prevention strategies. Using the same dealer was not considered an effective strategy to reduce overdose risk because of the uncertainty in the drug supply at every level, which has been reported in another study among people who have survived opioid overdose [26]. Tester shots or slow shots were acceptable and considered particularly important if fentanyl could be a possible contaminant and after periods of not using opioids (eg, incarceration). Fentanyl test strips were generally viewed as helpful but were considered unavailable at the time we conducted the focus groups. Studies have shown fentanyl test strips to be acceptable, easy to use, and associated with overdose prevention behaviors (eg, not using drugs that test positive for fentanyl and having naloxone available) [31]. As the lack of a relative safety benefit from smoking fentanyl, compared with injecting it, has become apparent, we have tailored risk reduction messages regarding route of administration to the opioid being used, and we do not explicitly recommend smoking fentanyl for overdose prevention.

In addition to opioid overdose prevention strategies, we discussed effective overdose response. Participants had experience responding to overdose, including using naloxone, which was considered an effective and acceptable response strategy. Participants also described witnessing overdose reversals when stimulation was used (eg, applying something cold). Study participants considered these alternative response strategies as effective, which has been reported in another study among people who use opioids [32]. For naloxone or other overdose response strategies to be effective, there must be someone present who is able to respond. Of the 10 participants, 3 (30%) reported using opioids alone, and they described strategies to remain accessible to others in the event that they experienced an overdose. In a study among people who recently used nonmedicinal opioids in New York City, New York, United States, age of ≥50 years and non-Hispanic Black race were associated with increased odds of not having naloxone present and a person trusted to administer it when using opioids [33]. Messages highlighting the importance of not using opioids alone should be emphasized among individuals who are less likely to have someone present if they overdose, which may include older and non-Hispanic Black people who use opioids.

There was a common fear of arrest or other negative consequences related to calling 911 in the event of an overdose, a common finding from prior research [25,34-41] that does not seem to have been significantly mitigated by Good Samaritan legislation. Techniques to minimize negative outcomes were discussed by participants, including telling the dispatcher that the person was “not breathing” and not that they had used drugs, which aligns with overdose response guidance from the National Harm Reduction Coalition [42]. Providing individuals with information about what to communicate to dispatchers may alleviate some fears associated with calling 911.

The subsequent phases of the ADAPT-ITT process, including expert review and testing, further helped to refine the intervention. These later modifications were typically minor, including additional detail to improve clarity and changing the manual’s formatting to increase usability. After internal testing at the study sites, we finalized the REBOOT 2.0 counseling manual and are currently evaluating the efficacy of the intervention in our ongoing clinical trial.

### Limitations

This study includes limitations. The focus groups were small, which can limit the generalizability of findings; however, considering the narrow and clear scope of the research (ie, adapting an existing intervention), active participation of the attendees, and repeat themes that arose during conversation, we believe that the number of attendees (n=10) was sufficient for the aims of this study [43]. Our findings may have been affected by social desirability and selection biases [40,44]. Nonetheless, our findings are similar to those among other groups of people who use opioids [29,30]. The study was conducted in a single setting, limiting the generalizability of findings to other locations. However, theater testing in the ADAPT-ITT framework is intended to be conducted among the population that the intervention is being adapted for, which in this case was survivors of opioid overdose who use opioids in Boston [15]. As fentanyl has since become more prevalent across the United States, our findings may have become relevant to other locations across the country.

Of the 10 participants, 3 (30%) were women, and additional research may be needed to further explore potential gender-specific intervention adaptations [39,45]. We requested feedback from 3 topical experts and our study teams, and their opinions may not reflect those of other experts and service providers of people who use opioids. Our focus group interview guide asked broad questions about participants’ perceptions of the counseling intervention and did not elicit feedback for each step of the intervention. More targeted questions could have provided more detailed recommendations for how to improve each counseling step.

### Conclusion

We successfully used the ADAPT-ITT approach to modify an opioid overdose prevention intervention, ensuring its applicability in a new geographic area and in the setting of a more potent street opioid. We conducted theater testing with people who use opioids, a challenge given the short-acting nature of fentanyl. Important adaptations resulted from this stage of the process, which led to a more appropriate intervention for people who use fentanyl. Incorporating input and lessons learned from people who use substances was key to optimizing our counseling intervention and messaging. Additional improvements were made through expert review and internal piloting. As fentanyl became the dominant street opioid in San Francisco during the full REBOOT trial, these adaptations proved essential.

## Appendix

Multimedia Appendix 1 Focus group guide.

Multimedia Appendix 2 Focus group handouts.

## Abbreviations

ADAPT-ITT: assessment, decision, adaptation, production, topical experts, integration, training, and testing
MOUD: medications for opioid use disorder
REBOOT: repeated-dose behavioral intervention to reduce opioid overdose
SKOOP: Skills and Knowledge on Overdose Prevention

## References

[1] (2021). Understanding the Epidemic. Centers for Disease Control and Prevention.

[2] Ciccarone D (2019). The triple wave epidemic: supply and demand drivers of the US opioid overdose crisis. Int J Drug Policy.

[3] Zoorob M (2019). Fentanyl shock: the changing geography of overdose in the United States. Int J Drug Policy.

[4] Ahmad FB, Cisewski JA, Rossen LM, Sutton P (2022). Provisional drug overdose death counts. National Center for Health Statistics.

[5] Mattson CL, Tanz LJ, Quinn K, Kariisa M, Patel P, Davis NL (2021). Trends and geographic patterns in drug and synthetic opioid overdose deaths - United States, 2013-2019. MMWR Morb Mortal Wkly Rep.

[6] Bennett AS, Bell A, Tomedi L, Hulsey EG, Kral AH (2011). Characteristics of an overdose prevention, response, and naloxone distribution program in Pittsburgh and Allegheny County, Pennsylvania. J Urban Health.

[7] Walley AY, Xuan Z, Hackman HH, Quinn E, Doe-Simkins M, Sorensen-Alawad A, Ruiz S, Ozonoff A (2013). Opioid overdose rates and implementation of overdose education and nasal naloxone distribution in Massachusetts: interrupted time series analysis. BMJ.

[8] Maxwell S, Bigg D, Stanczykiewicz K, Carlberg-Racich S (2006). Prescribing naloxone to actively injecting heroin users: a program to reduce heroin overdose deaths. J Addict Dis.

[9] Lankenau SE, Wagner KD, Silva K, Kecojevic A, Iverson E, McNeely M, Kral AH (2013). Injection drug users trained by overdose prevention programs: responses to witnessed overdoses. J Community Health.

[10] Wheeler E, Jones TS, Gilbert MK, Davidson PJ, Centers for Disease Control and Prevention (CDC) (2015). Opioid overdose prevention programs providing naloxone to laypersons - United States, 2014. MMWR Morb Mortal Wkly Rep.

[11] Coffin PO, Maya S, Kahn JG (2022). Modeling of overdose and naloxone distribution in the setting of fentanyl compared to heroin. Drug Alcohol Depend.

[12] Strang J, McDonald R, Alqurshi A, Royall P, Taylor D, Forbes B (2016). Naloxone without the needle - systematic review of candidate routes for non-injectable naloxone for opioid overdose reversal. Drug Alcohol Depend.

[13] Bohnert AS, Bonar EE, Cunningham R, Greenwald MK, Thomas L, Chermack S, Blow FC, Walton M (2016). A pilot randomized clinical trial of an intervention to reduce overdose risk behaviors among emergency department patients at risk for prescription opioid overdose. Drug Alcohol Depend.

[14] Coffin PO, Santos GM, Matheson T, Behar E, Rowe C, Rubin T, Silvis J, Vittinghoff E (2017). Behavioral intervention to reduce opioid overdose among high-risk persons with opioid use disorder: a pilot randomized controlled trial. PLoS One.

[15] Wingood GM, DiClemente RJ (2008). The ADAPT-ITT model: a novel method of adapting evidence-based HIV interventions. J Acquir Immune Defic Syndr.

[16] Staton M, Knudsen HK, Walsh SL, Oser C, Pike E, Lofwall M (2021). Adaptation of a standard extended-release naltrexone (XR-NTX) protocol for rural re-entering offenders with OUD. Health Justice.

[17] Cochran G, Gordon AJ, Field C, Bacci J, Dhital R, Ylioja T, Stitzer M, Kelly T, Tarter R (2016). Developing a framework of care for opioid medication misuse in community pharmacy. Res Social Adm Pharm.

[18] Coffin PO, Tracy M, Bucciarelli A, Ompad D, Vlahov D, Galea S (2007). Identifying injection drug users at risk of nonfatal overdose. Acad Emerg Med.

[19] Piper TM, Rudenstine S, Stancliff S, Sherman S, Nandi V, Clear A, Galea S (2007). Overdose prevention for injection drug users: lessons learned from naloxone training and distribution programs in New York City. Harm Reduct J.

[20] Somerville NJ, O'Donnell J, Gladden RM, Zibbell JE, Green TC, Younkin M, Ruiz S, Babakhanlou-Chase H, Chan M, Callis BP, Kuramoto-Crawford J, Nields HM, Walley AY (2017). Characteristics of fentanyl overdose - Massachusetts, 2014-2016. MMWR Morb Mortal Wkly Rep.

[21] (2018). The Massachusetts Opioid Epidemic: An Issue of Substance. Massachusetts Taxpayers Foundation.

[22] Pardo B, Taylor J, Caulkins JP, Kilmer B, Reuter P, Stein BD (2019). Understanding America's Surge in Fentanyl and Other Synthetic Opioids. RAND Corporation.

[23] Rev.

[24] Glaser BG (1965). The constant comparative method of qualitative analysis. Grounded Theory Rev.

[25] Bowles JM, Smith LR, Verdugo SR, Wagner KD, Davidson PJ (2020). "Generally, you get 86'ed because you're a liability": an application of Integrated Threat Theory to frequently witnessed overdoses and social distancing responses. Soc Sci Med.

[26] Kahn LS, Wozniak M, Vest BM, Moore C (2022). "Narcan encounters:" overdose and naloxone rescue experiences among people who use opioids. Subst Abus.

[27] Urmanche AA, Beharie N, Harocopos A (2022). Fentanyl preference among people who use opioids in New York City. Drug Alcohol Depend.

[28] McLean K, Monnat SM, Rigg K, Sterner 3rd GE, Verdery A (2019). "You never know what you're getting": opioid users' perceptions of fentanyl in Southwest Pennsylvania. Subst Use Misuse.

[29] Rowe C, Santos GM, Behar E, Coffin PO (2016). Correlates of overdose risk perception among illicit opioid users. Drug Alcohol Depend.

[30] Behar E, Chang JS, Countess K, Matheson T, Santos GM, Coffin P (2019). Perceived causes of personal versus witnessed overdoses among people who inject opioids. Subst Use Misuse.

[31] Goldman JE, Waye KM, Periera KA, Krieger MS, Yedinak JL, Marshall BD (2019). Perspectives on rapid fentanyl test strips as a harm reduction practice among young adults who use drugs: a qualitative study. Harm Reduct J.

[32] Bennett AS, Freeman R, Des Jarlais DC, Aronson ID (2020). Reasons people who use opioids do not accept or carry no-cost naloxone: qualitative interview study. JMIR Form Res.

[33] Bennett AS, Scheidell J, Bowles JM, Khan M, Roth A, Hoff L, Marini C, Elliott L (2022). Naloxone protection, social support, network characteristics, and overdose experiences among a cohort of people who use illicit opioids in New York City. Harm Reduct J.

[34] Latimore AD, Bergstein RS (2017). "Caught with a body" yet protected by law? Calling 911 for opioid overdose in the context of the Good Samaritan Law. Int J Drug Policy.

[35] Wagner KD, Harding RW, Kelley R, Labus B, Verdugo SR, Copulsky E, Bowles JM, Mittal ML, Davidson PJ (2019). Post-overdose interventions triggered by calling 911: centering the perspectives of people who use drugs (PWUDs). PLoS One.

[36] Davidson PJ, Ochoa KC, Hahn JA, Evans JL, Moss AR (2002). Witnessing heroin-related overdoses: the experiences of young injectors in San Francisco. Addiction.

[37] Tracy M, Piper TM, Ompad D, Bucciarelli A, Coffin PO, Vlahov D, Galea S (2005). Circumstances of witnessed drug overdose in New York City: implications for intervention. Drug Alcohol Depend.

[38] Durieux J, Curtis A, Mirka M, Jefferis E, Felix C, Essel B (2022). An exploration of Narcan as a harm reduction strategy and user's attitudes toward law enforcement involvement in overdose cases. Int J Environ Res Public Health.

[39] Ataiants J, Mazzella S, Roth AM, Sell RL, Robinson LF, Lankenau SE (2021). Overdose response among trained and untrained women with a history of illicit drug use: a mixed-methods examination. Drugs (Abingdon Engl).

[40] Rochester E, Graboyes M (2022). Experiences of people who use drugs with naloxone administration: a qualitative study. Drugs (Abingdon Engl).

[41] van der Meulen E, Chu SK, Butler-McPhee J (2021). "That's why people don't call 911": ending routine police attendance at drug overdoses. Int J Drug Policy.

[42] (2020). Opioid overdose basics: responding to an opioid overdose. National Harm Reduction Coalition.

[43] Morse JM (2000). Determining sample size. Qual Health Res.

[44] Latkin CA, Edwards C, Davey-Rothwell MA, Tobin KE (2017). The relationship between social desirability bias and self-reports of health, substance use, and social network factors among urban substance users in Baltimore, Maryland. Addict Behav.

[45] Harris MT, Bagley SM, Maschke A, Schoenberger SF, Sampath S, Walley AY, Gunn CM (2021). Competing risks of women and men who use fentanyl: "the number one thing I worry about would be my safety and number two would be overdose". J Subst Abuse Treat.

