# Supplementary material for: “There’s No Heroin Around Anymore. It’s All Fentanyl.” Adaptation of an Opioid Overdose Prevention Counseling Approach to Address Fentanyl Overdose: Formative Study
Source: JMIR Form Res. 2022 Sep 7;6(9):e37483. doi: 10.2196/37483 (PMC9494212; doi:10.2196/37483)
Supplement: Multimedia Appendix 1 [file formative_v6i9e37483_app1.docx]

**REBOOT ADAPT-ITT Focus Group Interview Guide**

**SCHEDULE OF EVENTS**

- **Participant Arrival** (*15 minutes*)
  - Participants arrive and a staff member hands them a copy of the verbal consent form for them to review. Research Assistants will be available to answer any questions about the process and the consent form.
  - Participants will be asked their name or preferred name to use on a tent card to be place in front of them at the table.
- **Introductions** *(2 minutes)*
  - The focus group facilitator will introduce themselves and the additional observers (Boston and/or SF research staff members).
  - The facilitator will review the goals and ground rules for the focus group.
  - The facilitator will lead a brief icebreaker.
- **Video viewing and discussion** *(1.5 hours)*
  - Participants will view the video and participate in a facilitator-led discussion.
  - A brief 10 minute break will be held at the halfway point.

**INTRODUCTION & GROUND RULES**

**Facilitator Script:** *Thank you all so much for agreeing to participate in our focus group today. I first want to go over a few important points from the verbal consent you all reviewed when you arrived.*

*The purpose of this focus group is to get feedback from you on a counseling session that deals with opioid overdose risk and prevention. We are going to have you view a video of one of the counselling sessions and then ask you what you think of it. Your responses, ideas, and suggestions are very valuable to us. You are the ones with the most experience on this topic and your insights will help us in planning for the larger study.*

*Now I want to review a few items from the consent form you just read:*

1. *Today’s meeting is going to be audio-recorded. This recording will only be reviewed by members of our research team so we can learn how to improve this counselling session.*
2. *What we talk about today is private and needs to stay within this room. Also, please do not share with others outside this room the names or identities of your fellow participants. In the event that you recognize anyone in the video, please do not reveal that person’s identity to the group.*

*Any questions about this?*

*Lastly, I want to review some ground rules for our discussion today, in order to ensure that this is a safe space for feedback.*

1. *Like I said before, anything said here, stays here.*
2. *Let’s all take turns in speaking and try not to talk over people. Because we want to hear from everyone, I might call on someone specifically to share if we’re hearing a lot from one person. If you don’t want to share, that’s ok too. Feel free to pass on a question.*
3. *It’s okay to disagree. Everyone has their own opinions, but please be respectful of the opinions of others.*
4. *We will have a break halfway through, but if you need to use the restroom, just grab one of our research staff outside and they will lead you.*
5. *Please turn off your cell phone and put them away so that they don’t disturb the group.*

*Are there any rules you have questions about or others you’d like to add?*

**INTRODUCTION TO VIDEO**

**Facilitator Script:** *Before we get started with the video of the counselling session, I just want to remind you all that this intervention was created and recorded San Francisco. The goal of today’s discussion is hear from you what we can do to make this work for people in Boston. The person who is counselled in this video may have a different story and experience from you, but what I want you to focus on is what the counselor asks her and the overall structure of the conversation.*

**~START VIDEO~**

**ICEBREAKER**

**Facilitator Script:** *To start things off, I have a few quick questions for the group. Please raise your hand if you’ve ever carried naloxone/narcan* rescue kit

*Please raise your hand if you’ve ever given narcan to someone.*

*Thank you everyone for sharing. Now let’s talk about the video we just watched together.*

**DISCUSSION**

**Content:**

1. What information in the counseling session was new for you? What information was not new for you?
2. What aspects of the counseling session were particularly helpful? Why?
   1. What parts were the most important or stood out to you the most?
3. What aspects of the counseling session were not as helpful? How could we make these more helpful?

**Drug Use Questions:**

1. Knowing that fentanyl is often present in the drug supply, what if anything, do you do differently when using?
   1. How should we adapt the intervention to account for this?
2. How relevant was the session’s content to your life and your daily experiences?
3. What are the ways that the counseling session could have better addressed your own experiences, specifically in regards to using fentanyl?
4. Is there anything else that you think is important for us to know about reducing the risk for overdose (or about working with people who inject drugs)?
5. How helpful, if at all, was the exercise activity (coming up with a personal plan to reduce overdose risk)? How could it be improved?

**General Feedback:**

1. What helps you remember things best? Seeing or hearing information? Participating in activities?
   1. How helpful would it be to see some of the information presented on laminated cards so that you could read along with the counselor?
2. As a potential participant in this conversation, do you think this discussion would have held your interest?
3. How long do you think these sessions should be?
4. What do you think might encourage participants to attend these sessions?
   1. What would get in the way of you coming to these sessions?
