# Supplementary material for: “There’s No Heroin Around Anymore. It’s All Fentanyl.” Adaptation of an Opioid Overdose Prevention Counseling Approach to Address Fentanyl Overdose: Formative Study
Source: JMIR Form Res. 2022 Sep 7;6(9):e37483. doi: 10.2196/37483 (PMC9494212; doi:10.2196/37483)
Supplement: Multimedia Appendix 2 [file formative_v6i9e37483_app2.pdf]

Behaviors that Contribute to Overdose Risk

- Using more or stronger opioids
- Having overdosed before
- Mixing opioids with other drugs like benzos, alcohol, or cocaine
- Injecting
- Using alone (including in a bathroom by yourself, even if there are others inside the building) so no one is there to help you if you do overdose
- Using different opioids or opioids from a different connection or dealer
- Not doing a tester
- Relying on others to prepare your drugs and/or to inject you
- Using the same amount the day you leave detox, treatment, jail, or the hospital as the day you went into detox, treatment, jail or the hospital. (It is really important to do a tester after any period of days that you haven't used opioids).
- Optional: Using fentanyl, even if you know that you are using it (as fentanyl is about 4 times riskier for overdose than injecting heroin)

Ways to Reduce Overdose Risk

- Avoid using other drugs with opioids.
- Use a smaller amount after a break/treatment/incarceration.
- Do a tester before doing the normal dose. (It is really important to do a tester after any period of days that you haven't used opioids).
- Use only while someone is right there, physically present with you, and who is equipped with narcan/naloxone and a phone to call for help, if necessary.
- Take turns, do not all use at the same time, so someone is awake in case it's a strong drug.
- Make sure someone who could help you has access to narcan/naloxone.
- Always carry narcan/naloxone and let others know where you keep it. Know where others keep their narcan/naloxone.
- Use fentanyl test strips on drugs before using to determine whether they may be laced with fentanyl
- Learn to prepare your own drugs
- Learn how to inject yourself
- Reduce use or quit, and/or seek substance use treatment.

3 Steps in the Management of Witnessed Overdose

- **Step 1**: **Recognize an overdose**
  - Someone has lost consciousness after using drugs, is turning blue, or is not breathing normally.
  - Use sternal rub to attempt to awaken the individual.
  - Do a "Verbal Narcan": Tell the person that you are planning to use naloxone to rescue them: "If you can't wake up, I will use Naloxone to rescue you!"
- **Step 2**: **Respond to the overdose**
  - Contact 911.
  - Provide rescue breathing and/or chest compressions.
  - Administer naloxone (as soon as it is available):
    - Always carry naloxone (or at least know where it is for quick access)
    - Should have new naloxone every two years because it expires.
    - Remember, naloxone only lasts about 1 hour, so the effects of the opioid they used may come back and person may overdose again.
    - Review criteria for a second dose of naloxone.
  - Fentanyl may cause a faster overdose – where the person's heart stops sooner – so you may need to do chest compressions right away, an ambulance may be needed sooner, and naloxone might need to be given several times.
- **Step 3**: **Provide after-care**
  - Place person in recovery position.
  - Stay with the person until help arrives or they are awake for at least 2 – 3 hours (longer if they were using prescription opioids).
  - After naloxone is provided, encourage the person not to use again for at least 1 hour.
